# Intergenerational impact of religious abuse on anorexia nervosa

**DOI:** 10.1111/ped.70245

**Published:** 2025-10-16

**Authors:** Shunichiro Nakamura

**Affiliations:** ^1^ Department of Pediatrics Keio University School of Medicine Tokyo Japan

**Keywords:** anorexia nervosa, child abuse, intergenerational transmission, neglect, religious abuse

In 2022, Japan's Agency for Children and Families defined religious abuse, but no reports exist on its intergenerational impact in Japan.^1^ This paper examines its effects through a case of anorexia nervosa. (Modified for privacy with patient consent.)

A 15‐year‐old Japanese girl with no significant medical and family history presented with an 11 kg weight loss over 6 months. Her height was 150.3 cm and weight was 33.2 kg (BMI 14.7 kg/m^2^, Z‐score −3.5). She had a strong fear of obesity and body image distortion, meeting DSM‐5 criteria for anorexia nervosa. She lived with her mother and 12‐year‐old sister. Her parents divorced when she was seven, and she lost contact with her father. Bullying triggered her dieting, but her mother ignored her distress, suggesting psychological neglect. One month before the presentation, the mother and the patient's sister went on a trip, leaving the patient alone at home for 3 weeks. As she told no one, child protection did not intervene. On her first visit, we advised better nutrition and reduced activity. Frustrated by the patient's refusal to eat, the mother stopped supervising meals. Continued weight loss led to hospitalization 4 months later. The mother's religious beliefs were unknown at the time. The mother's religious beliefs did not reject medical care or attribute illness to spiritual causes. However, her community praised children who stayed home alone as “good” for enduring while parents attended religious duties. A religious member knew the patient was alone for 3 weeks but took no action.

Upon admission, mother‐daughter visits ceased. The patient struggled to express emotions or symptoms, reflecting severe self‐esteem deficits. A week later, an article with a religious leader's words was found in clothing from her mother, revealing the mother's deep religious ties.

The mother reported suffering religious abuse from her parents, who forced her into religious activities with threats of violence and shaved her head. They often left her alone for religious duties, and her marriage was arranged by the community (Figure 1a). Although she resented her parents, she replicated similar parenting. Since the patient was two, the mother left her and her sister alone every weekend for religious gatherings. When the patient refused religious activities, the mother ignored her and took only the sister on outings and trips. Food shortages were common due to religious financial contributions. Additionally, the mother's proselytizing among the patient's friends' families led to her isolation at school. The patient stated, “For my mother, religion mattered more than me.” She believed, “If I have no value, I must stay thin,” damaging her self‐esteem and reinforcing her restrictive eating. Her BMI reached 21 after 2 years (Figure 1b,c), when they finally met again. Even after over 200 sessions, the mother continued criticizing her, often lashing out: “I don't want to talk to you.” She dismissed her daughter and the pediatrician, repeating her religious leader's words. This reflected rejection of public support and rigid religious adherence. The patient was informed that her mother's behavior was unlikely to change and that she had to leave the pediatric ward. After 3.5 years of hospitalization, she agreed to be discharged at 18 on the condition that she would not be forced into religious activities and returned home. She received independent and employment support immediately after discharge. As expected, psychological and financial abuse resumed, leading her to move out and begin living alone a year later.

**FIGURE 1:**
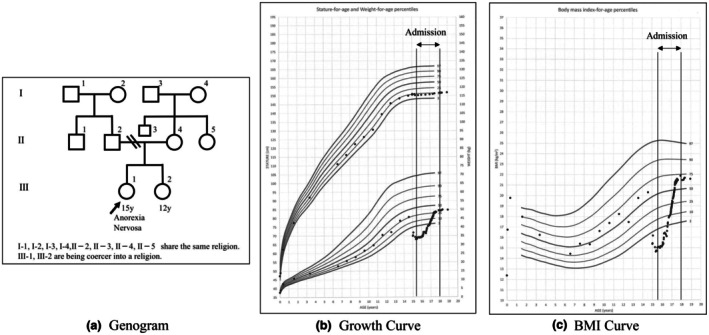
This patient's genogram, Growth curve and BMI curve. (a) This patient's genogram. I‐1, I‐2, I‐3, I‐4, II‐2, II‐3, II‐4, II‐5 share the same religion. III‐1, III‐2 are being coerced into a religion. (b, c) This patient's growth and BMI curves for patients, with vertical lines marking the start (left line) and end (right line) of hospital stays.

In this case, the mother experienced religious abuse, which was perpetuated across generations. Intergenerational transmission is more common in closed environments where religious values are absolute, often fostering adult dependence on authority.^2^ Raised in such a setting, the mother prioritized religion over caregiving and continued the cycle of abuse by leaving her child unattended. There was no external intervention in her parenting. It reflects a closed system where outside help is unlikely.

Victims of religious abuse face elevated risks of attachment disorders, impaired decision‐making, depression, and anxiety. In this case, the patient—who refused religious activities—was rejected and ignored by her mother, fostering a deep sense of worthlessness.^3^, ^4^, ^5^

Because religious abuse occurs in closed settings, early detection is difficult. In anorexia nervosa, clinicians should consider whether religious beliefs may be contributing factors.

## AUTHOR CONTRIBUTIONS

S.N. was solely responsible for designing the study, performing treatment, collecting and analyzing data, and writing the manuscript. S.N. also reviewed and approved the final manuscript.

## FUNDING INFORMATION

This study received no specific funding.

## CONFLICT OF INTEREST STATEMENT

Shunichiro Nakamura hereby declares that I have no financial or non‐financial interests to disclose in relation to the manuscript being submitted. We confirm that there are no personal or direct financial benefits that could potentially influence the outcomes of this research.

## DISCLOSURE

The authors have no financial relationships relevant to this article to disclose.

## INFORMED CONSENT

Written informed consent was obtained from the patient for publication of this case report.

## Data Availability

The data that support the findings of this study are available from the corresponding author upon reasonable request.
